# Second-line therapy for *Helicobacter pylori* eradication causing antibiotic-associated hemorrhagic colitis

**DOI:** 10.1186/s12941-017-0230-0

**Published:** 2017-08-14

**Authors:** Kazuyuki Tanaka, Mikihiro Fujiya, Aki Sakatani, Shugo Fujibayashi, Yoshiki Nomura, Nobuhiro Ueno, Shin Kashima, Takuma Goto, Junpei Sasajima, Kentaro Moriichi, Toshikatsu Okumura

**Affiliations:** Division of Gastroenterology and Hematology/Oncology, Department of Medicine, Asahikawa Medical University, 2-1 Midorigaoka-higashi, Asahikawa, Hokkaido 078-8510 Japan

## Abstract

**Objective:**

*Helicobacter pylori* (*H. pylori*) eradication rarely develops into antibiotic-associated hemorrhagic colitis (AAHC), in which the etiology of colitis remains unclear. We herein report a rare case of AAHC caused by second-line therapy for *H. pylori* eradication.

**Results:**

A 65-year-old female was administered second-line therapy for *H. pylori* composed of 1500 mg of amoxicillin, 500 mg of metronidazole and 40 mg of vonoprazan for 7 days because of first-line therapy failure. A day after completing second-line therapy, she complained of abdominal pain and hematochezia. Colonoscopy revealed a hemorrhage and edematous mucosa with no transparent vascular pattern in the transverse colon. A bacterial culture detected *Klebsiella oxytoca* (*K. oxytoca*), but no other pathogenic bacteria. A drug-induced lymphocyte stimulation test (DLST) showed positive reactions for both amoxicillin and metronidazole. According to these findings, the patient was diagnosed with AAHC. Bowel rest for 6 days relieved her abdominal pain and hematochezia.

**Conclusions:**

The present case developed AAHC caused by second-line therapy for *H. pylori* eradication. The pathogenesis is considered to be associated with microbial substitution as well as a delayed-type allergy to antibiotics, suggesting that AAHC is a potential adverse event of second-line therapy for *H. pylori* eradication.

## Background

*Helicobacter pylori* (*H. pylori*) infection has been known to cause many gastrointestinal disorders including gastroduodenal ulcers, gastric cancer and lymphoma, and idiopathic thrombocytopenic purpura [1–5]. *H. pylori* eradication is thus recommended to prevent these gastrointestinal disorders [6, 7]. In Japan, treatment for *H. pylori*-associated gastritis is now covered by health insurance [8]. The success rate of first-line therapy for *H. pylori* eradication is considered to be around 70–80% [9]. Second-line therapy for *H. pylori* eradication is needed for the remaining cases. Although *H. pylori* eradication occasionally causes adverse events, such as diarrhea and eruptions, it rarely develops into antibiotic-associated hemorrhagic colitis (AAHC), in which the etiology of colitis remains unclear. We herein report a rare case of AAHC caused by second-line therapy for *H. pylori* eradication.

## Case report

**Fig. 1:**
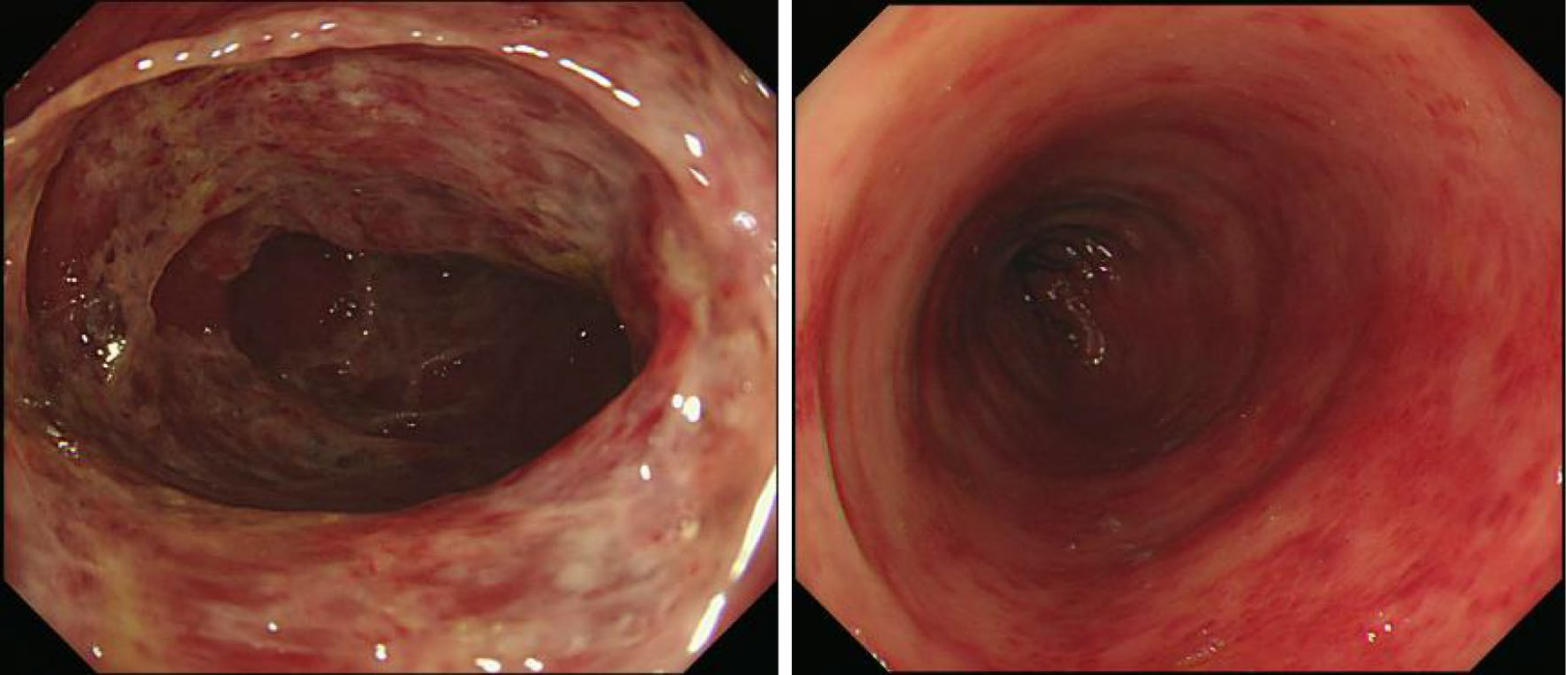
Colonoscopy findings. Diffusely edematous mucosa with mucus, a hemorrhage and no transparent vascular pattern was detected in the transverse colon

**Fig. 2:**
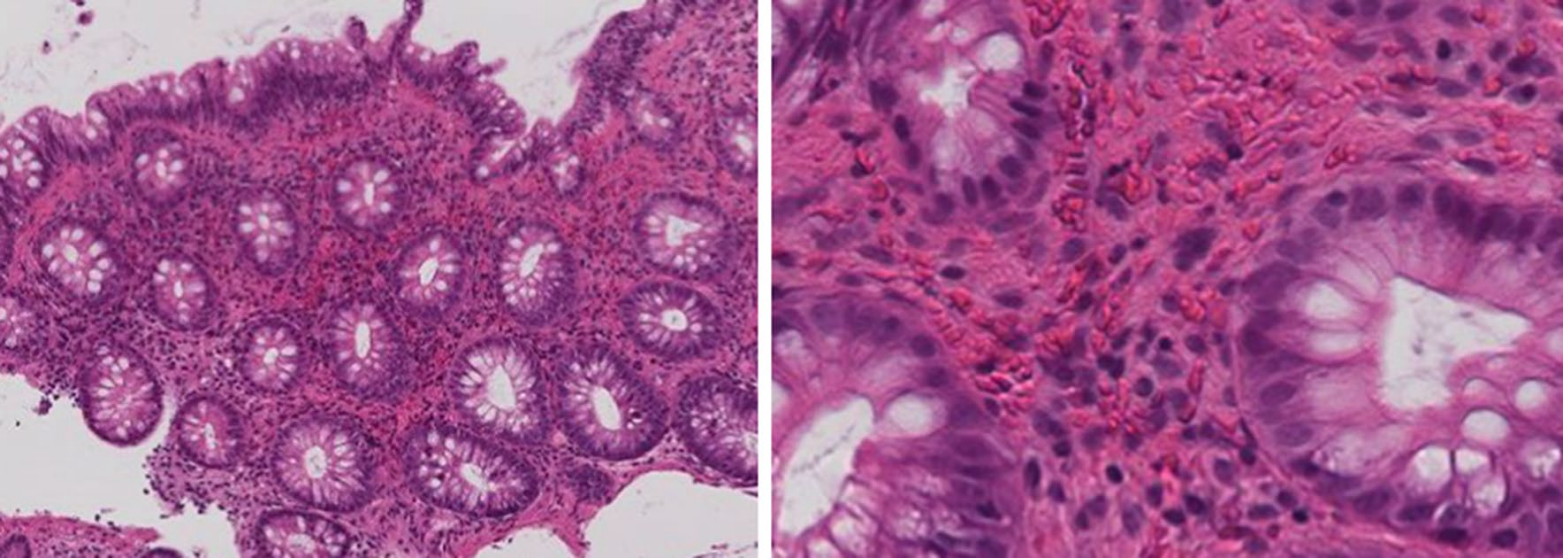
Histological findings. Severe infiltrations of neutrophil and lymphocytes and a hemorrhage in the lamina propria were detected in the biopsy specimens obtained from the transverse colon (magnifications: 100/400)

A 65-year-old female, who had systemic lupus erythematosus and was taking 5 mg of prednisolone, underwent gastric mucosal resection to remove gastric adenoma. The endoscopic findings of chronic gastritis were confirmed. Histological findings and culture of the gastric specimens obtained from the gastric antrum and body by an endoscopic biopsy procedure detected an *H. pylori* infection. She was therefore administered first-line therapy composed of 1500 mg of amoxicillin, 800 mg of clarithromycin and 60 mg of lansoprazole for 7 days to eradicate *H. pylori* in December 2014. A urea breath test was performed to assess the effect of the eradication and revealed a positive result for *H. pylori* infection. Seven months after the first-line therapy, the patient was prescribed second-line therapy composed of 1500 mg of amoxicillin, 500 mg of metronidazole and 40 mg of vonoprazan for 7 days to eradicate *H. pylori* in July 2015. A day after completing second-line therapy, she complained of abdominal pain and hematochezia. Blood tests showed increases in neutrophil (6090/µL) and C-reactive protein (1.38 µg/mL) levels, whereas the hemoglobin level was within the normal limits. Colonoscopy revealed a hemorrhage and edematous mucosa with no transparent vascular pattern in the transverse colon (Fig. 1). Histological findings of the biopsy specimens obtained from the transverse colon showed severe infiltrations of neutrophil and lymphocytes and a hemorrhage in the lamina propria (Fig. 2). A bacterial culture detected *Klebsiella oxytoca* (*K. oxytoca*), but not *Clostridium difficile* or other pathogenic bacteria. A drug-induced lymphocyte stimulation test (DLST) showed positive reactions for both amoxicillin and metronidazole. According to these findings, the patient was diagnosed with AAHC.

Bowel rest for 6 days under the oral administration of 5 mg of prednisolone relieved her abdominal pain and hematochezia. Typical symptoms due to SLE have been relieved for 20 months with conservative treatment alone, and no vasculitis was histologically detected in biopsy specimens of the colon. Therefore, lupus enteritis was not thought to be associated with the pathogenesis of the enteric disorder in the patient.

## Discussion

We herein report a rare case of AAHC that developed after the completion of second-line therapy using amoxicillin, metronidazole and vonoprazan for *H. pylori* eradication. Clinicians must pay careful attention to the possibility of this disorder, even when no side effects develop after the first-line therapy for *H. pylori* eradication.

While the incidence rate of AAHC after the first-line therapy for *H. pylori* eradication has been reported to range from 0.35 to 0.6% [10, 11], the frequency of AAHC after second-line therapy for *H. pylori* eradication is still unclear. A total of five previous cases that exhibited AAHC after completing second-line therapy for *H. pylori* eradication were identified in a literature search on the PubMed and Ichushi databases (Table 1) [12, 13]. Including the present case, there were 2 males and 4 females, and the mean age of the cases was 59.3 years old. Five cases exhibited AAHC after completing second-line *H. pylori* eradication therapy, suggesting that an immediate-type allergy was not a cause of AAHC. In two cases, *K. oxytoca* was isolated from the stool culture. It is known that stool testing reveals *K. oxytoca* in significant amounts in the majority of patients with AAHC [14]. *K. oxytoca* strains isolated from patients with AAHC are known to produce a cytotoxin, which were shown to cause cell death in cultured Hep2, Vero, CHOK1, and HeLa cell lines as well as in an isolated intestinal loop rabbit model [15, 16]. These studies indicated that microbial substitution by amoxicillin- and/or metronidazole-induced overgrowth of *K. oxytoca* could be associated with the development of AAHC in patients with the administration of second-line therapy for *H. pylori* eradication. The DLST was performed only in our case, which detected positive reactions for amoxicillin and metronidazole. Collectively, two mechanisms are thought to be involved in the pathogenesis of AAHC. First, microbial alterations accumulate by repeated antibiotic administration, thereby leading to AAHC at the second-line treatment. Second, amoxicillin sensitization may have been acquired at the time of first-line therapy, and a delayed allergic reaction may have occurred at the time of second-line therapy. Although the delayed-type allergy was a potential cause of AAHC in our case, it is unclear whether this type of allergy is a major cause of AAHC in patients receiving second-line therapy for *H. pylori* eradication. A greater accumulation of cases is warranted to understand the etiology of AAHC due to second-line therapy for *H. pylori* eradication.

**Table 1:** Reported cases of antibiotic-associated hemorrhagic colitis due to the second-line therapy for Helicobacter pylori eradication

Age	Gender	Symptoms	Onset	First-line therapy	Second-line therapy	Fecal bacterial culture	DLST	References
58	F	Abdominal pain, bloody diarrhea	8 days after starting eradication	CAM + AMPC + RPZ	MNZ 500 mg + AMPC 1500 mg + RPZ 20 mg	Pathogenic bacteria was not detected	Not described	#12
54	M	Abdominal pain, bloody diarrhea	7 days after starting eradication	CAM + AMPC + RPZ	MNZ 500 mg + AMPC 1500 mg + RPZ 20 mg	Pathogenic bacteria was not detected	Not described	#12
59	F	Abdominal pain, bloody diarrhea	9 days after starting eradication	CAM + AMPC + RPZ	MNZ 500 mg + AMPC 1500 mg + RPZ 20 mg	Pathogenic bacteria was not detected	Not described	#12
60	F	Abdominal pain, bloody diarrhea	9 days after starting eradication	CAM + AMPC + PPI	MNZ + AMPC + PPI	*Klebsiella oxytoca*	Not described	#13
50	M	Abdominal pain, hematochezia	6 days after starting eradication	CAM + AMPC + PPI	MNZ + AMPC + PPI	Not described	Not described	#13
65	F	Abdominal pain, hematochezia	8 days after starting eradication	CAM 800 mg + AMPC 1500 mg + LPZ 60 mg	MNZ 500 mg + AMPC 1500 mg + Vonoprazan 40 mg	*Klebsiella oxytoca*	Positive for both AMPC and MNZ	Present case

*CAM* clarithromycin, *AMPC* amoxicillin, *MNZ* metronidazole, *RPZ* rabeprazole, *LPZ* lansoprazole, *PPI* proton pump inhibitor

## Conclusions

In summary, we herein reported a rare case of AAHC caused by second-line therapy for *H. pylori* eradication. Due to the detection of *K. oxytoca* and positive reaction on the DLST for amoxicillin and metronidazole, the pathogenesis of the present case was considered to be associated with microbial substitution as well as a delayed-type allergy to amoxicillin and/or metronidazole. Clinicians must pay careful attention to the possibility of AAHC, even when administering second-line therapy for *H. pylori* eradication.

## Abbreviations

*H. pylori*: *Helicobacter pylori*
AAHC: antibiotic-associated hemorrhagic colitis
*K. oxytoca*: *Klebsiella oxytoca*
DLST: drug-induced lymphocyte stimulation test
CAM: clarithromycin
AMPC: amoxicillin
MNZ: metronidazole
RPZ: rabeprazole
LPZ: lansoprazole
PPI: proton pump inhibitor
